# Peripheral retinal vessel whitening in patients with diabetes mellitus

**DOI:** 10.1038/s41598-023-35124-5

**Published:** 2023-05-17

**Authors:** Fritz Gerald P. Kalaw, Paripoorna Sharma, Rasha Nabil Kako, Evan Walker, Shyamanga Borooah

**Affiliations:** 1 Jacobs Retina Center, University of California San Diego, San Diego, CA 92093 USA; 2 Viterbi Family Department of Ophthalmology and Shiley Eye Institute, University of California San Diego, 9415 Campus Point Dr, La Jolla, San Diego, CA 92093 USA; 3 Division of Ophthalmology Informatics and Data Science, Viterbi Family Department of Ophthalmology and Shiley Eye Institute, University of California San Diego, San Diego, CA 92093 USA

**Keywords:** Prognostic markers, Endocrine system and metabolic diseases, Eye manifestations

## Abstract

This study aimed to identify retinal vessel whitening outside the standard Early Treatment Diabetic Retinopathy Study (ETDRS) fields and to correlate the findings with vision and severity of diabetic retinopathy. Patients with diabetes mellitus who were seen in the retinal clinic to assess diabetic retinopathy status were included. Retinal vessel whitening was identified using ultra-widefield imaging. Four hundred and forty-five eyes of 260 patients were included. Thirty-five eyes in 24 patients (7.9%) were noted to have peripheral retinal vessel whitening. Thirty-one eyes with peripheral retinal vessel whitening did not have vessel whitening within the standard 7 ETDRS fields (*p* < 0.001). The proportion of whitening increased as DR severity increased, from 4.0% for patients with no DR (OR 0.249) to 33.3% for those with severe NPDR and PDR (OR 6.430 and 7.232, respectively). In addition, patients with peripheral retinal vessel whitening had worse visual acuity (logMAR = 0.34) compared to those without (logMAR = 0.15) (*p* < 0.001). In conclusion, we found an association between peripheral retinal vessel whitening in diabetic patients which correlated with diabetic retinopathy severity. Additionally, we found an association between vessel whitening and reduced vision, suggesting that vessel whitening identified using ultra-widefield imaging may be a prognostic indicator of vision in diabetic retinopathy.

## Introduction

According to the 2019 report by the Centers for Disease Control and Prevention (CDC), 96 million people (38.0% of the adult population aged 18 years or older) have prediabetes, and 37.3 million people (11.3% of the population) have diabetes mellitus (DM) in the United States alone^1^. Vascular complications of both the macro- and microvascular systems are the leading causes of morbidity and mortality in individuals with DM^2^. Diabetic retinopathy (DR) is the most common DM complication and one of the leading causes of preventable blindness in the adult working population^3^. Over 4 million US adults aged 40 years and older have diabetic retinopathy (1 in 29), with around 900 thousand persons having vision-threatening diabetic retinopathy (VTDR) (1 in 132)^4^. In 2020, the number of adults with DR and VTDR was estimated to be 103.12 million and 28.54 million globally, respectively^3^.

Over 50 years ago, several leaders in the field of ophthalmology, internal medicine, and neurosurgery gathered to discuss and develop a comprehensive system to describe diabetic retinopathy and its treatment modalities. Color photographic standards were also established to describe retinal findings – hemorrhages, hard exudates, venous abnormalities, and neovascular and fibrous proliferation. The images were localized in the seven standard stereoscopic photographic fields, concentrating on the posterior 90 degrees of the retina. This is the known Airlie House Classification of diabetic retinopathy^5^. A modification was used in the Diabetic Retinopathy Study (DRS) and Early Treatment Diabetic Retinopathy Study (ETDRS) and has become a gold standard for many years. This modified classification classifies DR into 13 complex levels, an excellent tool in research; however, it has had limited clinical application^6^. To simplify the classification, Wilkinson and colleagues proposed the International Clinical Diabetic Retinopathy and Diabetic Macular Edema Disease Severity Scales. This used five disease severity levels: no apparent retinopathy, mild non-proliferative diabetic retinopathy (NPDR), moderate NPDR, severe NPDR, and proliferative diabetic retinopathy (PDR)^7^. This classification has been used widely by clinicians, perhaps from its simplified severity definition.

The introduction of ultra-widefield (UWF) retinal imaging enables capturing images from as wide as 200-degree field of view in just a fraction of a second. This helps identify and localize lesions and pathologies in the retinal periphery that can be difficult to obtain using a regular 30-degree fundus camera that may also need manual steering of the optical piece. More recently, UWF imaging has been studied in DR. Domalpally and colleagues compared the DR severity scale outside the standard 7-field images using UWF imaging. Their group noted an 8.3% (13/156 eyes) increase in the DR severity when peripheral images (referred to in their work as “global ETDRS imaging”) were used^8^.

Retinal microvascular changes include microaneurysms, hemorrhages (characterized as flame-shaped or blot, depending on the retinal layer affected), intraretinal microvascular abnormality, venous beading, exudate, neovascularization, and vessel whitening. All these findings were characterized in the clinical severity grading of DR and diabetic macular edema (DME)^7^, except for vessel whitening. Retinal vessel whitening represents a final common pathway for several hypoxic processes and is thought to be a sequela of an ischemic event. The retinal vessels appear white due to the accumulation of material within the vascular lumen and resultant vascular wall ischemia, resulting in vascular endothelial cell death^9^. Development of retinal vessel whitening can occur in retinal arterioles and venules from vascular occlusion or thrombosis, respectively^9^. Retinal vessel whitening has also been associated with retinal arterial or vein occlusion (BRAO, BRVO), which can lead to visual loss.

The association between retinal vessel whitening and diabetes has not been widely studied. Our study aimed to identify retinal vessel whitening in DM patients using a UWF scanning laser ophthalmoscope (SLO) and to understand its relationship to diabetic retinopathy grading and vision.

## Methods

### Design

This study was approved by the Institutional Review Board of the University of California San Diego (IRB # 120516). Data collection and analysis were conducted according to the Principles of the Declaration of Helsinki and complied with the Health Insurance Portability and Accountability Act (HIPAA) of 1996. The patients provided written informed consent as per institution protocol, and all data were anonymized for patients’ safety.

### Study participants, examinations, and procedures

Participants in the study were consecutive DM type 1 and 2 patients who were seen and diagnosed by retinal specialists in the retinal outpatient clinic at the University of California San Diego from September 15, 2020 until December 29, 2021 for new screening or as established patients. Only the first visit image of patients who had multiple visits in the timeframe was used in the study, and both eyes were included when available. Participants underwent comprehensive ophthalmologic examination, including best corrected visual acuity (BCVA) using Early Treatment Diabetic Retinopathy Study (ETDRS) visual acuity chart, intraocular pressure, slit lamp biomicroscopy, and dilated fundus examination using a binocular indirect ophthalmoscope. Eyes were graded by retinal specialists in clinic as having no apparent retinopathy, mild non-proliferative, moderate non-proliferative, severe non-proliferative, or proliferative diabetic retinopathy. Pseudocolor imaging was obtained using the UWF Optos California P200DTx (Optos plc, Dunfermline, UK). Patients with media opacity from the cornea, lens, or vitreous, which prevented clear visualization of the retina, patients who have had prior retinal laser procedures (barricade laser, focal laser, or panretinal photocoagulation), or retinal diseases which may induce whitening of retinal vessels (central or branch retinal occlusion) were excluded from the study.

### Ultra-widefield image processing

Images captured from the Optos system were downloaded and transferred securely from the OptosAdvance imaging database as .jpeg for image processing. Manual interpolation of the standard 7-field ETDRS rings was provided per image using Microsoft Powerpoint (version 16.58, Microsoft Corporation, Redmond WA) (Supplementary Figure S1), and all images were saved in one document and viewed on a single computer screen.

### Retinal vessel whitening identification

**Figure 1:**
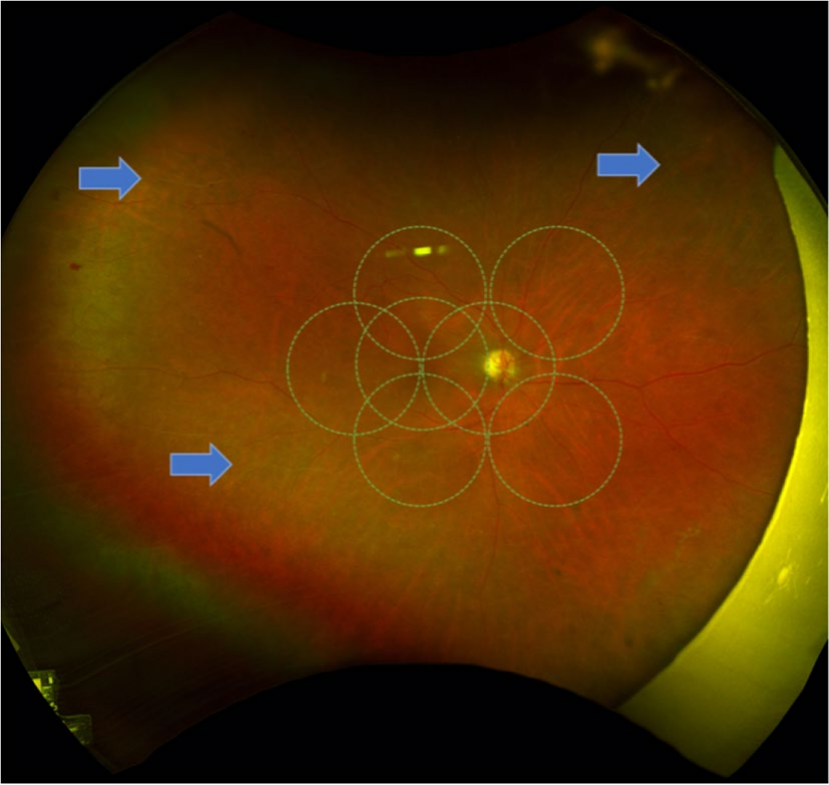
Representative sample of a patient with Moderate Non-Proliferative Diabetic Retinopathy with peripheral retinal vessel whitening (*arrows*) at the superotemporal, superonasal, and inferotemporal arcades.

**Figure 2:**
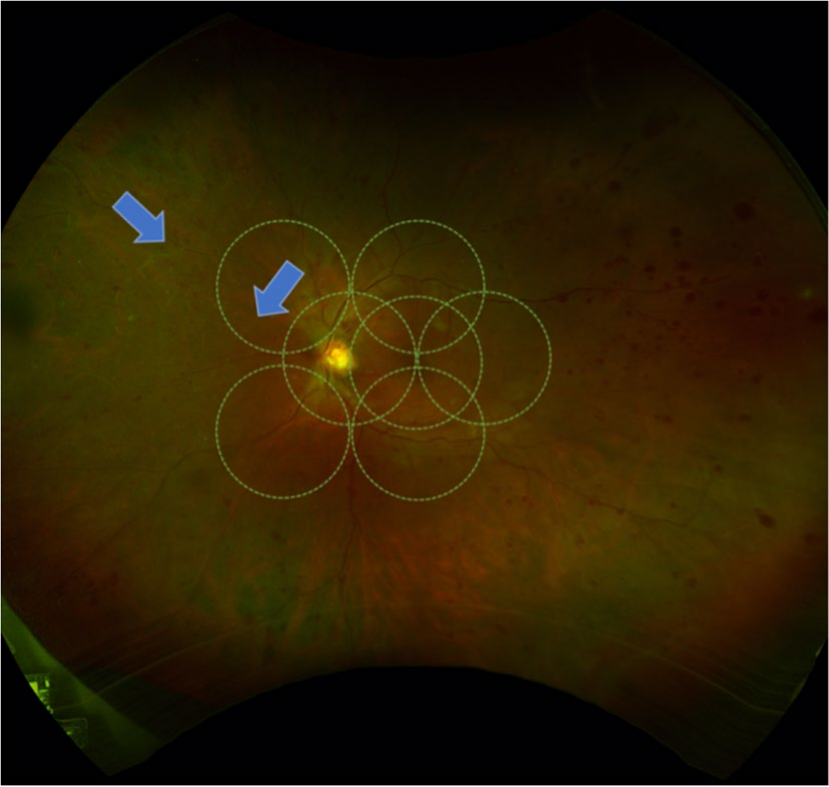
Representative sample ultra-widefield pseudocolor image from a patient with Proliferative Diabetic Retinopathy with peripheral retinal vessel whitening within and outside the 7 ETDRS Fields (*arrows*).

**Figure 3:**
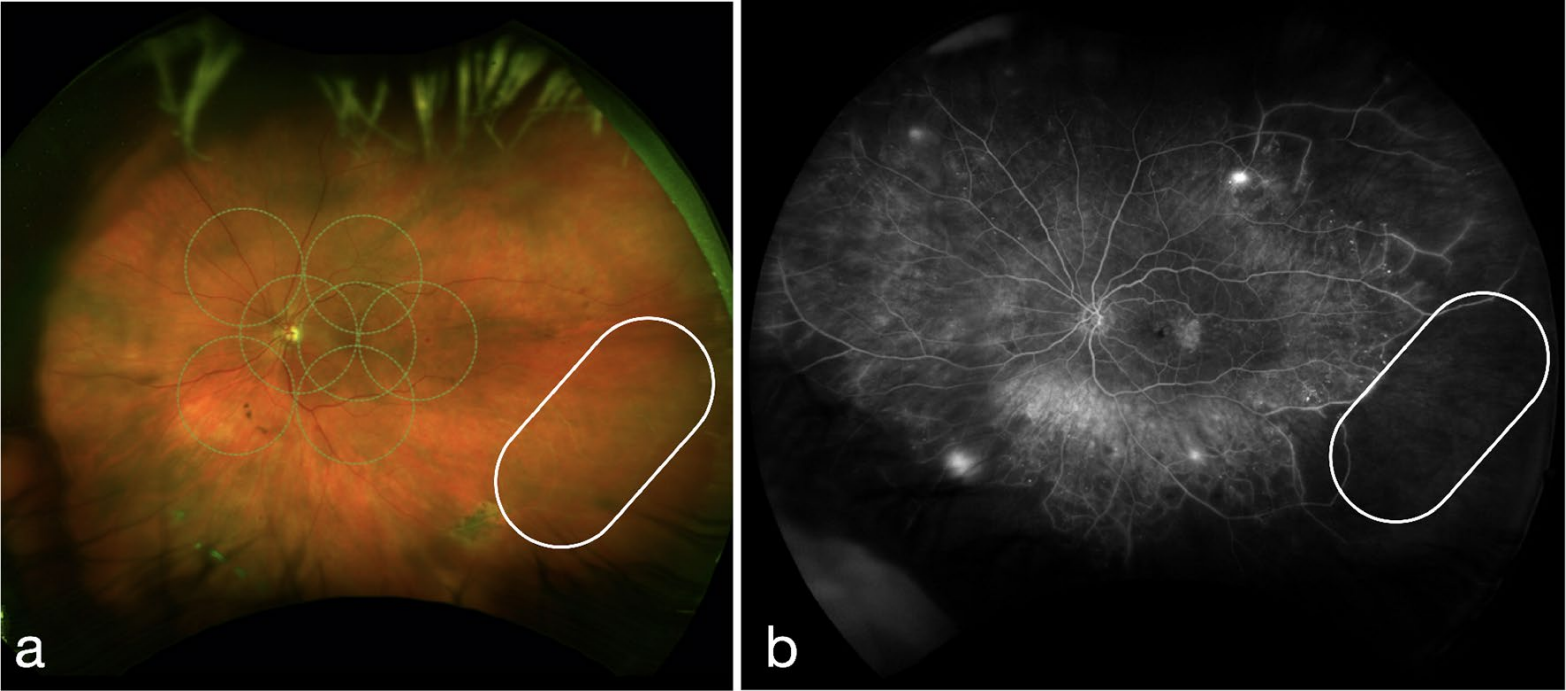
Representative ultra-widefield pseudocolor (**a**) and fluorescein angiogram (**b**) images from a patient with Proliferative Diabetic Retinopathy with Diabetic Macular Edema showing capillary non-perfusion and vascular filling defect within the areas of peripheral retinal vessel whitening (encircled).

Two experienced ophthalmologists acted as independent graders to identify retinal vessel whitening. The graders were asked to identify any whitening of the retinal vessel within and outside the interpolated ETDRS rings and identify the vessel affected (whether an arteriole or venule). (Figs. 1 and 2) In patients with identified retinal vessel whitening, available fundus fluorescein angiography (FFA) was obtained and inspected for capillary non-perfusion and vascular filling defects in the area of vessel whitening. (Fig. 3).

### Statistical analysis

Demographic and clinical characteristic data were presented as count (%) for categorical variables and mean (95% confidence interval) for continuous variables. Statistically significant differences between cohorts were determined using t-tests and Fisher’s Exact tests, for subject-level continuous and categorical characteristics, respectively, and linear mixed-effects models were used to address the use of two eyes from patients. Given the smaller sample size of the subjects with peripheral retinal vessel whitening cohort, parametric assumptions were confirmed through Shapiro-Wilks, Kolmogorov–Smirnov, and F-tests. Additionally, Fisher’s Exact test was used to evaluate pairwise comparisons of individual categorical characteristics between vessel whitening cohorts. Statistical analyses were performed using the R programming language version 4.2.1 (R Foundation for Statistical Computing, Vienna, Austria). A *P*-value ≤ 0.05 was considered statistically significant.

## Results

A total of 294 diabetic patients were identified. Five-hundred twelve eyes were initially assessed. None of the patients was identified with a history of central or branch retinal vein occlusion. Sixty patients had imaging consistent with a prior retinal laser procedure, and seven patients had poor ocular media, and hence were excluded. To validate the inter-observer grading, the two independent graders were initially given 30 eyes to observe for whitening of retinal vessels. Cohen’s Kappa was used to assess the agreement between the graders’ findings, which revealed a value of 0.625, classified as substantial agreement.

In total, 445 eyes of 260 patients were included in the study. Thirty-five eyes in 24 patients (7.9%) were noted to have peripheral retinal vessel whitening. (Fig. 1) Table 1 shows the demographic characteristics of patients with and without peripheral retinal vessel whitening. The age, sex, and DM type were not statistically significantly different between the two groups.

**Table 1:** Subject and Eye Level Demographic and Clinical Differences between Subjects With and Without Peripheral Retinal Vessel Whitening.

	Without peripheral retinal vessel whitening (n = 236 subjects)	With peripheral retinal vessel whitening (n = 24 subjects)	*p*-value
Age (mean [CI])	62.4 (60.6, 64.3)	62.8 (57.0, 68.6)	0.905
Sex			1.000
F (%)	116 (49.2%)	12 (50.0%)	
M (%)	120 (50.8%)	12 (50.0%)	
DM Type			0.713
1 (%)	22 (9.3%)	3 (12.5%)	
2 (%)	214 (90.7%)	21 (87.5%)	
BCVA (logMAR mean [CI])	0.15 (0.13, 0.18)	0.34 (0.26, 0.41)	< 0.001*
Race			0.009*
African American or Black (%)	13 (5.5%)	5 (20.8%)	
Asian (%)	63 (26.7%)	2 (8.3%)	
Hispanic (%)	26 (11.0%)	5 (20.8%)	
Other or Mixed Race (%)	26 (11.0%)	4 (16.7%)	
White (%)	108 (45.8%)	8 (33.3%)	
DR Severity Grading			< 0.001*
No DR (%)	266 (64.9%)	11 (31.4%)	
Mild NPDR (%)	80 (19.5%)	6 (17.1%)	
Moderate NPDR (%)	40 (9.8%)	6 (17.1%)	
Severe NPDR (%)	8 (2.0%)	4 (11.4%)	
PDR (%)	16 (3.9%)	8 (22.9%)	
ETDRS 7-field Whitening			< 0.001*
No	408 (99.5%)	31 (88.6%)	
Yes	2 (0.5%)	4 (11.4%)	

* Bold *p*-values denote statistical significance of less than 0.05.

*BCVA* Best-corrected visual acuity, *logMAR* Logarithm of Minimum Angle of Resolution, *DR* Diabetic retinopathy, *NPDR* Non-proliferative diabetic retinopathy, *PDR* Proliferative diabetic retinopathy, *ETDRS* Early treatment diabetic retinopathy study.

Patients with peripheral retinal vessel whitening were noted to have a lower BCVA (0.34, equivalent to 68 letter score or 20/40-20/50 Snellen chart) compared to those without whitening (0.15, equivalent to around 77 letter score or 20/25-20/32 Snellen chart) (*p* < 0.001). In order to try to exclude the confounding effect of diabetic macular edema (DME) on BCVA, we performed further analysis in patients without DME across both cohorts. Twenty (20/35) and three-hundred fifty-one (351/410) eyes without DME were identified from those with and without peripheral retinal vessel whitening, respectively. After accounting for DME, the patients with peripheral retinal vessel whitening still had a significantly lower BCVA (*p* = 0.0236) with a mean BCVA of 0.26 (equivalent to 72 letter score or 20/32-20/40 Snellen chart) when peripheral retinal vessel whitening was present compared to 0.14 (equivalent to 78 letter score or 20/25-20/32 Snellen chart) when peripheral retinal vessel whitening was not present, suggesting that peripheral vessel whitening may be associated with poorer visual function.

The analysis also noted a statistically significant difference between the distribution of races among the two groups (*p* = 0.028). A post hoc analysis of race revealed that 63 out of 65 Asian patients with DM had not shown peripheral retinal vessel whitening (*p* = 0.045) (Table 2).

**Table 2:** Individual comparisons of Race between Peripheral Retinal Vessel Whitening Cohorts.

	Without peripheral retinal vessel whitening (n = 236 subjects)	With peripheral retinal vessel whitening (n = 24 subjects)	*p*-value
Race			
African American or Black (%)	13 (5.5%)	5 (20.8%)	0.067
Asian (%)	63 (26.7%)	2 (8.3%)	0.045*
Hispanic (%)	26 (11.0%)	5 (20.8%)	0.171
Other or Mixed Race (%)	26 (11.0%)	4 (16.7%)	0.999
White (%)	108 (45.8%)	8 (33.3%)	0.861

* bold *p*-value denotes statistical significance of less than 0.05.

To understand whether peripheral retinal vessel whitening correlated with the findings of the original DR grading, we checked the proportion of times retinal vessel whitening was seen with each DR severity grading subtype. The peripheral retinal vessel whitening was noted to have an increased odds ratio with DR severity grading (Table 3), suggesting that increasing peripheral retinal vessel whitening was associated with increasing severity of DR.

**Table 3:** Individual comparisons of DR Severity Grades between groups With and Without Peripheral Retinal Vessel Whitening.

	Without peripheral retinal vessel whitening (n = 410 eyes)	With peripheral retinal vessel whitening (n = 35 eyes)	Proportion of Whitening	Odds Ratio
DR Severity Grading				
No DR	266	11	4.0%	0.249
Mild NPDR	80	6	7.0%	0.854
Moderate NPDR	40	6	13.0%	1.910
Severe NPDR	8	4	33.3%	6.430
PDR	16	8	33.3%	7.232

*DR* Diabetic retinopathy, *NPDR* Non-proliferative diabetic retinopathy, *PDR* Proliferative diabetic retinopathy.

To better understand the distribution of whitened retinal vessels in DR patients, we looked to see how often the whitened vessels were seen in the standard 7-field ETDRS view. There was a statistically significant difference between the distribution of vessel whitening seen within or outside of the ETDRS 7-field region (*p* < 0.001). A post hoc analysis showed an 88.6% absence of whitening within the ETDRS 7-field region among the patients with peripheral retinal vessel whitening (*p* < 0.001) (Table 4). This suggests that the majority of retinal vessel whitening in diabetic patients may not be noted if the traditional ETDRS measurement is used.

**Table 4:** ETDRS 7-field Whitening in Eyes With Peripheral Retinal Vessel Whitening.

	Without ETDRS 7-field whitening	With ETDRS 7-field whitening	*p*-value
With Peripheral Whitening	31 (88.6%)	4 (11.4%)	< 0.001*

*ETDRS* Early treatment diabetic retinopathy study.

To understand the relationship between retinal ischemia and vessel whitening, we reviewed the images of those in our cohort who had undergone FFA. We identified 12 eyes from the 35 patients with peripheral retinal vessel whitening who have had FFA. Vascular filling defect, a type of abnormal hypofluorescence, occurs when the visible retinal vessel fails to emit the normal fluorescence pattern upon transit of the fluorescein dye. Capillary nonperfusion, another type of abnormal hypofluorescence, is the absence of an area of the capillary bed to fluoresce upon transit of the fluorescein dye. In our cohort, seven eyes had vascular filling defects, while eight had capillary non-perfusion, suggesting ischemia, although both of which did not show statistical significance (*p* = 0.683 and 0.221, respectively) (Table 5). Interestingly, both retinal arterioles and venules were noted to be affected (17 arteriolar, 14 venular, 4 both) in cases with peripheral retinal vessel whitening.

**Table 5:** Fundus Fluorescein Angiography Findings in the Area of Peripheral Retinal Vessel Whitening.

	Yes	No	*p*-value
Peripheral retinal vessel angiographic findings (n = 12 eyes)			
Vascular filling defect	7	5	0.683
Capillary non-perfusion	8	4	0.221

## Discussion

DR classifications have not included retinal vessel whitening in prior grading criteria. This may be partly due to the relative rarity of such findings in the standard central 7 ETDRS fields. However, the development of UWF imaging now makes review of the retinal periphery more accessible. Unsurprisingly, several studies have reviewed UWF imaging findings in DR patients and compared peripheral findings to the standard 7 ETDRS field findings, to assess for DR progression, and many have identified more extensive pathology, including proliferative changes in the periphery when compared to the standard 7 ETDRS field findings^8,10–13^. The established method of grading DR severity focuses primarily on the posterior pole, which captures only up to 35% of the retinal surface compared to the UWF imaging, which can capture up to 82% of the retinal surface, extending visualization of the retina^10^. Taken together, this suggests that DR grading using findings outside the standard 7 ETDRS field may be helpful to assist severity grading and, ultimately, the risk of vision loss from diabetes. The present study investigated another finding more commonly seen outside the standard 7 ETDRS field region in diabetic patients, retinal vessel whitening, using UWF imaging. Our group noted a 7.9% (35/445 eyes) prevalence of retinal vessel whitening, all of which were present outside the standard 7 ETDRS fields, with only four out of the 35 eyes having the concomitant presence of whitening within the standard 7 ETDRS fields. This finding suggests that the whole UWF imaging will need to be used to identify this finding.

Retinal whitening appears to have a functional significance for patients with diabetes. Patients with peripheral retinal vessel whitening were also noted to have significantly poorer BCVA than those without peripheral retinal vessel whitening, even when DME was excluded. This is perhaps not surprising, considering retinal vessel whitening is associated with poor retinal perfusion, as we have also confirmed in this study. However, it is perhaps surprising that peripheral changes may have an impact on central retinal changes. BCVA is usually driven by macular function. As a result, peripheral retinal vessel whitening may be an indicator of reduced global retinal perfusion. Mouse models exhibited structural (retinal thinning) changes as well as functional (vision loss) as blood glucose levels increased^14^. Investigation of the macular retinal architecture and optical coherence tomography angiography among patients with peripheral retinal vessel whitening may provide insight into the process of microvascular changes and visual function.

In the present study, peripheral retinal vessel whitening showed an increasing odds ratio with DR severity and was seen in as much as a third of patients with severe NPDR and PDR. This finding may also contribute to the limited published literature studying peripheral UWF imaging findings in the context of DR progression.

Various researchers have analyzed the relationship between retinal vascular caliber and geometry and DR severity^15–19^. Although these studies have revealed associations between vascular findings and DR severity, performing these measurements would entail utilizing computer-assisted software to measure retinal vessel changes that would necessitate time and experienced programming to track certain differences. Our group posed a simple yet straightforward evaluation of a retinal feature that appears to correlate with DR severity without needing further analysis tools.

There are a few limitations to our study. The current study had a relatively small number of more progressive DR (i.e., severe NPDR and PDR). A higher number and variety of participants that reflect the real-world population will be beneficial to assess the possible use of this finding. The present study analyzed fundus photographic images of diabetic patients. Cross-sectional scans using UWF optical coherence tomography (OCT)^13^ and panoramic OCT angiography (OCTA)^20^ may provide additional insight into retinal layers and vasculature status that could aid in prognostication. These are planned as future studies. In addition, since this was a retrospective study from clinic data, grading may be limited by the clinical interpretations made by different retinal specialists.

In conclusion, studies in our cohort of diabetic patients suggest that identifying peripheral retinal vessel whitening using UWF imaging may assist in managing patients with diabetes as the clinical presentation of retinal vessel whitening appears to be associated with increasing DR severity as well as being a marker of poorer visual function.

## Supplementary Information

Supplementary Figure S1.

## Data Availability

All data in the current study are available from the corresponding author (SB) upon reasonable request.
