# Supplementary figures and images for: Peripheral retinal vessel whitening in patients with diabetes mellitus
Source: Sci Rep. 2023 May 17;13:7981. doi: 10.1038/s41598-023-35124-5 (PMC10192220; doi:10.1038/s41598-023-35124-5)

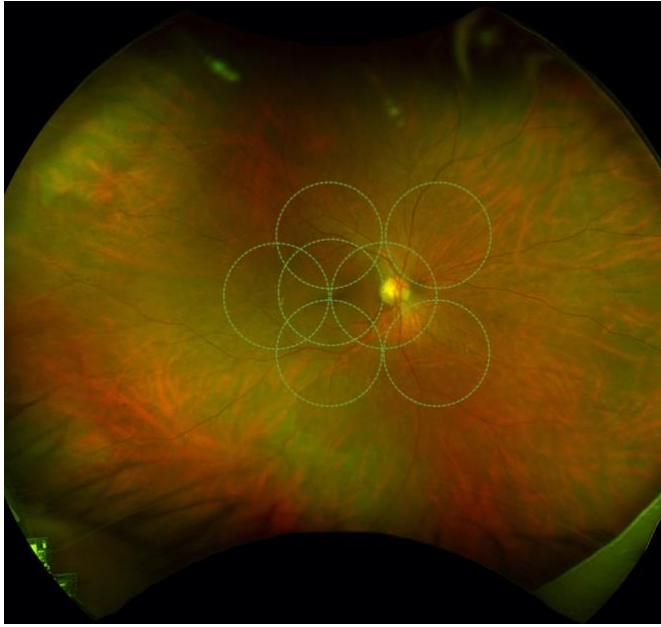

**Figure S1. Manually interpolated 7 Early Treatment Diabetic Retinopathy Study (ETDRS) Fields.**

Supplement: Supplementary file 1 — Supplementary Figure S1. [file 41598_2023_35124_MOESM1_ESM.pdf]
